# Spatio-temporal variation in territory quality and oxidative status: a natural experiment in the Seychelles warbler (*Acrocephalus sechellensis*)

**DOI:** 10.1111/j.1365-2656.2010.01792.x

**Published:** 2011-05

**Authors:** Janske van de Crommenacker, Jan Komdeur, Terry Burke, David S Richardson

**Affiliations:** 1 Animal Ecology Group/Behavioural Ecology and Self-Organisation Group, Centre for Ecological and Evolutionary Studies, University of GroningenPO Box 11103, 9700 CC, Groningen, The Netherlands; 2 Department of Animal and Plant Sciences, University of SheffieldSheffield S10 2TN, UK; 3 Centre for Ecology, Evolution and Conservation, School of Biological Sciences, University of East AngliaNorwich NR4 7TJ, UK; 4 Nature SeychellesPO Box 1310, Victoria, Mahé, Seychelles

**Keywords:** adaptive response, antioxidants, foraging, natural population, oxidative stress, physical activity, seasonal fluctuations, stress, time of day, trade-offs

## Abstract

**1**.Fluctuations in the quality of the habitat in which an animal lives can have major consequences for its behaviour and physiological state. In poor-quality habitat with low food availability, metabolically intensive foraging activity is likely to result in increased generation of reactive oxygen species, while scarcity of food can lead to a weakening of exogenously derived antioxidant defences. The consequent oxidant/antioxidant imbalance may lead to elevated oxidative stress.

**2**.Although the link between food availability and oxidative stress has been studied in the laboratory, very little is known about this relationship in the wild. Here, we investigate the association between territory quality (measured through food availability) and oxidative stress in the Seychelles warbler (*Acrocephalus sechellensis*).

**3**.Seychelles warblers are insectivorous birds that inhabit a fixed feeding territory year round. Individuals experience profound and rapid local fluctuations in territory quality within these territories, owing to changing patterns of vegetation defoliation resulting from seasonal changes in prevailing wind direction and wind-borne salt spray.

**4**.As expected, oxidant generation (measured as reactive oxygen metabolites; ROMs) was higher when territory quality was low, but there was no correlation between territory quality and antioxidant capacity (OXY). The negative correlation between territory quality and ROMs was significant between individuals and approached significance within individuals, indicating that the pattern resulted from individual responses to environmental variation.

**5**.ROMs and OXY levels within individuals were positively correlated, but the relationship between territory quality and ROMs persisted after including OXY as a covariate, implying that oxidative stress occurs in low territory quality conditions.

**6**.Our results indicate that the oxidative stress balance of an individual is sensitive to relatively short-term changes in territory quality, which may have consequences for the birds’ fitness.

## Introduction

The habitats in which animals live are typically not static, and individuals have to respond effectively to environmental changes to maintain the stability of their inner environment (homoeostasis). Responses are manifested as physiological, morphological and behavioural modifications (Möstl & Palme 2002; Sohal 2002), which may, in turn, have profound effects on individual fitness (Romero 2004). Fluctuations in food supply are the rule in nature, and animals constantly have to respond by altering the amount of time and energy spent obtaining sufficient food. When food availability is low, individuals have to bear the unfavourable physiological and energetic repercussions that may accompany metabolically intensive foraging behaviours.

One of the important consequences of physical activity is the acceleration of oxidative metabolism (Davies *et al.* 1982; Leeuwenburgh & Heinecke 2001; Monaghan, Metcalfe & Torres 2009). Reactive oxygen species (also referred to as oxidants) are generated as by-products of oxygen consumption (Finkel & Holbrook 2000; Balaban, Nemoto & Finkel 2005) and cause damage to biological macromolecules (Beckman & Ames 1998; Pérez-Campo *et al.* 1998). Cumulative oxidative damage may eventually lead to accelerated ageing and the development of degenerative diseases (Harman 1956; Ames, Shingenaga & Park 1991; Beckman & Ames 1998; Finkel & Holbrook 2000; Furness & Speakman 2008). The antioxidant machinery, which includes a series of endogenous and exogenous compounds that neutralize oxidants, potentially enables individuals to avoid these harmful effects (Felton & Summers 1995; Halliwell & Gutteridge 1999; Krinsky & Yeum 2003). However, the allocation of resources to antioxidant systems is thought to incur costs itself as a result of the trade-off with other fitness-relevant investments such as reproduction, sexual signalling, immune function and growth (Alonso-Alvarez *et al.* 2008; Bizé*et al.* 2008; Monaghan, Metcalfe & Torres 2009; Nussey *et al.* 2009). The oxidant–antioxidant balance, and consequently the rate at which oxidative damage is generated when more oxidants are produced than can be neutralized, represents the oxidative stress level (Finkel & Holbrook 2000; Costantini & Verhulst 2009).

In captive animals experimentally forced to work harder to obtain food, increases in energetic parameters such as daily energy expenditure and basal metabolic rate have been observed (Bautista *et al.* 1998; Deerenberg *et al.* 1998; Wiersma, Salomons & Verhulst 2005). Although the link between metabolic rate and oxidative stress is not straightforward (Barja 2007; Hulbert *et al.* 2007), studies in humans (e.g., Ashton *et al.* 1999; Ji 1999; reviewed in Leeuwenburgh & Heinecke 2001; Vollaard, Shearman & Cooper 2005) and captive animals (e.g., Davies *et al.* 1982; Magwere *et al.* 2006; Larcombe *et al.* 2008; reviewed in Monaghan, Metcalfe & Torres 2009) indicate a positive link between physical activity and oxidative stress. Moreover, flight effort has been shown to increase oxidative stress levels in birds and insects (Costantini, Cardinale & Carere 2007; Costantini, Dell'Ariccia & Lipp 2008; Williams, Agarwal & Elekonich 2008).

The availability of food can also affect antioxidant levels in animals, as diet-derived antioxidants play a substantial role in the total antioxidant defence system (Vertuani, Angusti & Manfredini 2004; Catoni, Peters & Schaefer 2008). Fruit, seed and invertebrate food sources all contain variable amounts of dietary antioxidants, and levels of circulating antioxidants in wild birds are generally positively related with the levels of antioxidants in their diet (see Cohen, McGraw & Robinson 2009). Poor food conditions can influence oxidative status by weakening dietary antioxidant defences (Catoni, Peters & Schaefer 2008), and supplementation of dietary antioxidants has been shown to increase total antioxidant capacity (De Ayala, Martinelli & Saino 2006; Cohen, Klasing & Ricklefs 2007; Catoni, Peters & Schaefer 2008). However, it is important to note that the low availability of dietary antioxidants may be compensated for by the upregulation of endogenously derived antioxidant defences (Selman *et al.* 2006; Monaghan, Metcalfe & Torres 2009).

Studies investigating the link between habitat quality (or food availability) and oxidative stress in the wild are now needed to help understand laboratory results in an ecological context, for example, to provide insight into the trade-off between investment in antioxidant defences and other fitness-relevant activities (Costantini 2008; Monaghan, Metcalfe & Torres 2009). Investigating ecophysiological links in natural model systems, in which individuals perform natural and voluntary behaviours, provides a valuable addition to laboratory studies that often do not take full notice of animals’ life histories, put animals in situations they have not evolved to deal with and are typically cross-sectional (reviewed in Monaghan, Metcalfe & Torres 2009).

Here, we investigate the link between territory quality and oxidative stress in a natural population of the Seychelles warbler (*Acrocephalus sechellensis*), a small passerine bird endemic to the Seychelles islands. As there is virtually no migration on or off the island (Komdeur *et al.* 2004), the Cousin Island population provides a closed study system. Seychelles warblers are purely insectivorous, taking 98% of their insect food from leaves (Komdeur 1991), and birds inhabiting year-round stable feeding territories often remain in the same territory throughout their lives (Komdeur 1992; Eikenaar *et al.* 2008). Individuals spend most of their time foraging (91% on average, Komdeur 1991), and there is a significant difference in the amount of time spent foraging between birds occupying different quality territories; the higher the territory quality, the less time spent foraging (Komdeur 1991, 1996).

On Cousin, territory quality is affected by changes in the direction of the prevailing monsoon winds (each 6 months, Dowling *et al.* 2001; Komdeur & Daan 2005). These short-term 180-degree shifts in wind direction have profound local effects on territory quality, particularly in the coastal territories where trees become defoliated because of onshore wind-driven salt spray (Dowling *et al.* 2001; Komdeur & Daan 2005). By taking advantage of these profound natural changes, our study aims to get a better understanding of how the birds cope with natural fluctuations in territory quality in terms of preserving their oxidative balance. To evaluate this balance, we measured both the oxidant component (measured as reactive oxygen metabolites, ROMs) and the antioxidant component (OXY) in the blood plasma of individuals. It is crucial to include both measures, as an assessment of only one part of the balance may lead to misinterpretation of the results (Monaghan, Metcalfe & Torres 2009). We predict a negative relationship between territory quality and ROMs as a result of the generation of oxidants when territory quality is low and foraging activity (metabolic activity) increases. We also expect a positive relationship between territory quality and OXY as a result of birds having access to more exogenous antioxidants when territory quality is high. Therefore, birds in low territory quality conditions are expected to have higher ROM levels as they work harder to obtain food and lower OXY as they gain less dietary antioxidants, both factors leading to higher oxidative stress. By taking a longitudinal approach (measuring the same individuals under different territory quality conditions over time), it is possible to distinguish between within-individual and between-individual effects in our analyses and to identify the extent to which the patterns observed result from individual responses. We expect the observed relationships, at least partially, to derive from within-individual effects, showing that birds individually respond to environmental changes in an attempt to maintain oxidative balance.

## Materials and methods

### Study area and population

Data were collected from the Seychelles warbler population on Cousin Island (29 ha; 04°20′S, 55°40′E), which has been studied intensively since 1985 (Komdeur 1991; Richardson, Komdeur & Burke 2003; Brouwer *et al.* 2010). From 1997 onwards, almost every individual has been individually ringed. Cooperative breeding occurs within this population (Komdeur 1992) with group sizes varying from 2 to 6 individuals (Komdeur 1994; Brouwer *et al.* 2006).

**Fig. 1:**
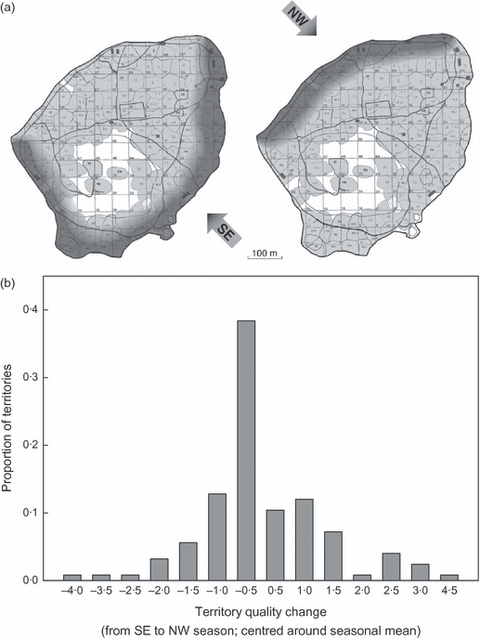
(a) Seychelles warbler territories on Cousin island with wind directions (arrows) during the SE (on the left) and NW season (on the right). The semi-annual 180-degree shifts in wind direction have profound local effects on territory quality, particularly in the coastal territories where trees become defoliated because of wind-driven salt spray (wind-affected coastal zones indicated with dark grey shading). Coastal zones located at the south side are of lower quality in the SE season than in the NW season, while the coastal zones located at the north side show the opposite pattern. Light grey areas are territories and white areas are rocks (no territories). (b) The frequency distribution of mean territory quality changes from the SE to the NW season over the years 2007–2008. To correct for yearly variation in territory quality, we centred the territory quality data around the seasonal mean.

Fieldwork was carried out in 2007 and 2008 during the main breeding season (July–September) in the south-east (SE) monsoon and the intermediate breeding period (January–March) in the north-west (NW) monsoon (Fig. 1a). In both seasons, all areas over the entire island were sampled. During the SE monsoon, strong prevailing winds (often force 5) come from the south-eastern direction, while in the NW monsoon they are generally lighter and less persistent (force 3–4) and come from the north-west (Fig. 1a). The changes in wind direction and wind-blown salt spray have profound local effects on defoliation (Dowling *et al.* 2001). Territories that are defoliated have been verified as having higher wind-blown NaCl (mg/l) concentrations (Dowling *et al.* 2001). Positive associations between salt exposure and territory quality may therefore be expected. Territories on the northern and southern coast differ in their pattern of territory quality fluctuations; territory quality conditions are rich on one shore and poor on the opposite shore, alternating when winds turn (Komdeur & Daan 2005). Birds were caught randomly in all locations on the island using mist nets that were checked at least every 15 min. This study focused on adult birds only – i.e., birds older than 8 months of age (the minimum age at which birds can produce young; Komdeur 1997).

### Assessment of territory quality and other environmental variables

As Seychelles warblers are insectivorous (Komdeur 1991), territory quality depends on the amount of insect prey within their territory. A territory quality index was calculated following Komdeur (1992, 1996) using the formula *a* × Σ(*c*_x_ *i*_x_), where *a* is the territory size (hectares), *c*_x_ is the foliage cover for broad-leafed tree species x, and *i*_x_ is the mean monthly insect count for tree species x per unit leaf area (dm^2^). Vegetation abundance was scored by determining the presence of 10 tree species at 20 random points in every territory on the island, in the following height bands: 0- to 0·75-m, 0·75- to 2-m and at 2-m intervals thereafter. Territory size was determined from territory maps constructed from detailed observational data of foraging and territorial defence behaviour by colour-ringed birds. Insect densities were estimated by monthly counting total insect numbers on the undersides of 50 leaves for each of 10 tree species present in 14 different regions across the island (Brouwer *et al.* 2009). These regions were based on the amount of defoliation caused by wind-driven salt spray (Komdeur 1991; Komdeur & Daan 2005; Brouwer *et al.* 2009). Insect counts taken in the most central territory in each region were used as an estimate for all territories within that region (Komdeur 1991).

The identity of all birds present in each territory was recorded. For each captured bird, the following variables were identified: (i) Territory quality; (ii) Sex; (iii) Age (in years): based on the long-term ringing data; (iv) Social status: based upon field observations. The ‘primary’ male and female were defined as the dominant, pair-bonded male and female in the territory. All other adult birds resident in the territory were defined as ‘subordinate’ (Richardson, Burke & Komdeur 2002), which could either be a ‘helper’ or a ‘non-helper’; (v) Group size: the number of adult individuals in the territory; (vi) Breeding activity: each territory was checked for nesting activity at least once every 2 weeks by following the resident female for 15 min (Komdeur 1992). Active nests were monitored throughout the breeding season to ascertain breeding stage; (vii) Time of day (in minutes since sunrise at 6·00 a.m.).

### Sampling

A blood sample (*c.* 100 μL) was taken from each bird immediately after catching by brachial venipuncture using heparinized capillary tubes. Samples were preserved from the ambient heat by placing them in a cool box filled with ice or, if nearby, in the fridge at the research station, directly after sampling. Approximately 80 μL of the sample was centrifuged at 9883 ***g*** for 8 min within 3 h of bleeding. The plasma was stored frozen (−18 °C) until further analyses. During transport, the samples were kept frozen using ColdSAFE Minus storage boxes (Absolute Cold, via Orbi-Solutions, Aalten, Netherlands) that allow for shipment in passenger aircrafts as no dry ice is involved. Storage temperatures were monitored continuously using data loggers (Verdict transport logging system; via Orbi-Solutions). The remaining blood was diluted in 1 mL of 100% ethanol in a screw-cap microfuge tube and stored at room temperature. DNA extracted from these samples (following Richardson *et al.* 2001) was used to confirm sex using the molecular method devised by Griffiths *et al.* (1998).

### Oxidative stress analyses

Oxidative damage compounds were measured using the d-ROMs test kit (Diacron, Grosseto, Italy) that quantifies ROMs – the products of free radical reactions with biological macromolecules. Specifically, the test measures the plasma concentration of hydroperoxides, a group of ROMs that are more stable and easier to detect than reactive oxygen species and are considered to be an accurate marker of oxidative damage to lipids and proteins (Alberti *et al.* 2000; Iamele, Fiocchi & Vernocchi 2002). Antioxidant capacity (OXY) was analysed using the OXY-Adsorbent test (Diacron), which measures the effectiveness of plasma antioxidants by quantifying its ability to cope with the oxidant action of hypochlorous acid (HClO). The test includes a large section of antioxidant compounds, such as exogenously (e.g., flavonoids and tocopherols) and endogenously (e.g., glutathione and bilirubin) synthesized antioxidants. Instructions provided with the kits were followed with a few minor modifications. In brief, 20 and 10 μL of plasma were used for the ROMs and OXY assays, respectively. Absorbencies were measured at 505 nm (spectrophotometer model DU-720; Beckman Coulter, Woerden, Netherlands). The ROMs are presented as mM of H_2_O_2_ equivalents and OXY as mM of HClO neutralized. Interassay variation was 2·25% (ROMs) and 1·85% (OXY), and intra-assay variation was 1·68% (ROMs) and 3·08% (OXY). A detailed description of the protocols can be found in Costantini & Dell'Omo (2006).

To ensure the reliability of sample quality and test results, the storage recommendations provided by the test kit manufacturer were followed closely. Previous studies showed that short- and long-term storage did not influence ROMs’ test results (Cavalleri *et al.* 2004; Pasquini *et al.* 2008), but one should note that the exact storage temperatures and durations were different from the conditions in our study. Our own analysis of both ROMs and OXY for goose aliquots that were stored in −18 °C for 6 months indicated that the storage procedures we used would not have been detrimental to the samples.

### Data analyses

Both dependent variables – ROMs and OXY – were normally distributed. The distribution of territory quality was positively skewed (skewness value: 2·07 ± 0·13), but achieved normality after being log-transformed. A subsample of individuals (*n* = 240) was measured repeatedly on different dates, which resulted in a data set comprising 339 observations (one individual measured 5 times, 3 individuals 4 times, 18 individuals 3 times, 49 individuals twice and 170 individuals only once). Only individuals that did not switch between territories within the study period were included, which resulted in the exclusion of 10 birds from the data set.

Hierarchical multilevel mixed models (MLWiN 2.13, Rasbash *et al.* 2004) were used with ‘territory’, ‘individual’ and ‘observation’ included as levels. ‘Assay’ (test session) was not included as a random term, because interassay variation was the same (ROMs) or lower (OXY) than the intra-assay variation (see preceding methods section). All explanatory variables that were fitted in the models are presented in Table 1. Season (2007 SE, 2007 NW, 2008 SE and 2008 NW seasons), breeding activity (pre-nesting = 0, nest-building = 1, incubating = 2 and provisioning = 3), sex (male = 0 and female = 1) and status (primary = 0, helper = 1 and non-helper = 2) were fitted as factors with ‘2007 SE’ and 0, respectively, chosen as reference categories. Time of day, age (range 1–14 years), group size and size-corrected body mass (residual between body mass and tarsus length, indicating body condition) were fitted as continuous variables. A second-order polynomial function of territory quality (territory quality^2^) was added to allow for the possibility of a quadratic relationship between the dependent factor and territory quality. This was also carried out for time of day and age. All interactions were tested but are only reported when statistically significant. Model selection was based on stepwise backward elimination of the non-significant terms in the order of their significance assessed by their Wald statistic. Significance level was set at *P* < 0·05. The final models (Table 1) contained the constant and all significant explanatory terms. All eliminated terms were reintroduced to the final model to confirm their lack of contribution and to check how they affected the fit of the model. As the inclusion of the size-corrected body mass resulted in a better fit of the model (but did not change the territory quality results), we included this in the final model.

**Table 1:** Model summary examining the effect of territory quality on (a) ROM levels and (b) OXY in the Seychelles warbler. Summaries derived from a normal response hierarchical mixed-modelling procedure. Terms left in the final model are shown in bold. Variances (with standard errors) are given for all random effects

	Estimate ± SE	χ^2^	d.f.	*P*
(a) ROMs				
Intercept	**2.38 ± 0.15**			
Territory quality (log)	**−0.18 ± 0.07**	**6.74**	1	**0.009**
Season^1^		**107.15**	3	**<0.001**
2007 NW season	**−**0.54 ± 0.08			
2008 SE season	**−**0.64 ± 0.07			
2008 NW season	**−**0.55 ± 0.08			
Time of day	**−0.001 ± 0.001**	**3.47**	**1**	**0.06**
Time of day (squared)	**<0.001 ± <0.001**	**7.06**	1	**0.008**
Breeding stage^2^		**9.52**	3	**0.023**
Nest-building	0.10 ± 0.07			
Incubating	**−**0.11 ± 0.08			
Provisioning	**−**0.13 ± 0.07			
Size-corrected body mass	**−0.06 ± 0.05**	**1.38**	**1**	**0.24**
Sex^3^	**−**0.06 ± 0.05	1.55	1	0.21
Status		0.37	2	0.83
Group size	0.02 ± 0.02	0.69	1	0.41
Age	<0.001 ± 0.01	0.00	1	0.99
Age (squared)	0.001 ± 0.002	0.19	1	0.66
Log territory quality (squared)	3.39 ± 3.51	0.94	1	0.33
Random effects				
σ_territory_^2^	0.00 ± 0.00	–	–	–
σ_individual_^2^	0.00 ± 0.00	–	–	–
σ_residual_^2^	0.18 ± 0.01	–	–	–

(b) OXY				
Intercept	**138.51 ± 3.55**			
Territory quality (log)	**−**2.80 ± 3.54	0.62	1	0.43
Season^1^		**47.19**	**3**	**<0.001**
2007 NW season	0.20 ± 3.40			
2008 SE season	**−**17.66 ± 3.16			
2008 NW season	**−**14.91 ± 3.41			
Time of day	**0.02 ± 0.01**	**5.24**	**1**	**0.022**
Sex^3^	**6.57 ± 2.88**	**5.23**	**1**	**0.022**
Status^4^		**5.26**	**2**	**0.07**
Helper	19.83 ± 8.79			
Non-helper	**−**0.62 ± 4.04			
Size-corrected body mass	**−0.10 ± 2.30**	**0.00**	**1**	**0.96**
Sex^3^ × Status^4^		**7.77**	**2**	**0.021**
Sex^3^ × helper	**−**16.43 ± 10.40			
Sex^3^ × non-helper	**−**14.36 ± 5.74			
Breeding stage		0.78	3	0.85
Group size	0.82 ± 1.29	0.40	1	0.53
Age	0.21 ± 0.44	0.22	1	0.64
Age (squared)	0.07 ± 0.12	0.35	1	0.55
Log territory quality (squared)	−76.05 ± 178.33	0.18	1	0.67
Time of day (squared)	<0.001 ± 0.001	0.02	1	0.9
Random effects				
σ_territory_^2^	57.07 ± 29.65	3.71	1	0.05
σ_individual_^2^	38.73 ± 44.95	0.74	1	0.39
σ_residual_^2^	345.85 ± 46.29	–	–	–

Reference categories: ^1^‘2007 SE season’. ^2^‘pre-nesting stage’. ^3^‘male’. ^4^‘primary’.

To separate within-individual effects from between-individual effects, the statistical within-subject centring procedure described by van de Pol & Wright (2009) was followed. Briefly, the mean territory quality for each individual across all observations was subtracted from the territory quality measured at each specific observation. This new predictor variable (the deviation in territory quality from the mean territory quality experienced by the individual) was used as a fixed effect expressing only the within-individual variation component (β_W_). The between-individual variation component (β_B_) was given by the mean territory quality for the individual across all observations. By splitting up the original fixed predictor effect from the final models of Table 1 into these two new fixed effects (β_W_ and β_B_), we tested whether either the within-individual effect or the between-individual effect was themselves significant. To investigate whether these two effects differed from each other, the original fixed predictor effect (i.e., territory quality) was again included alongside the new fixed between-individual variation (β_B_) predictor effect (thus leaving out β_W_). In this model, the between-individual effect now represented the difference between the between- and within-individual effects (β_B_ − β_W_). The estimate of β_B_ − β_W_ is expected to be non-significant when the within- and between-subject effects are effectively the same, thereby confirming that the original predictor variable is representative of within-individual effects and ensuring that inferences made about individual responses are not erroneously based solely on between-individual differences. The method is also used to check whether non-significant fixed effects are the result of the between- and within-individual effects going in opposite directions, thereby cancelling out each other.

A subset of birds that lived in coastal territories on opposing sides of the island was captured and re-captured in successive seasons under different season conditions. This allowed us to test (independent samples *t*-test) whether the change in seasons and the consequent change in territory quality conditions resulted in within-individual changes in residual ROMs (accounting for all factors present in the final model 1a) in the expected direction.

A bivariate general linear mixed model (GLMM) with both ROMs and OXY included as dependent variables was used to link territory quality with oxidative balance. This method has the ability to identify covariances across the response variables on the different grouping levels. The model (Table 2) included all explanatory variables that were left in the final models with ROMs and OXY separately as dependent variables (from Table 1a,b). As in the first models, size-corrected body mass was also included in the final model. Significance of the random terms was tested by performing likelihood ratio tests, except for the observation level that principally represents the unexplained variation. Significance of variances (for both ROMs and OXY) and covariances on the territory and individual level are reported in Table 2. In these likelihood ratio tests, a model with unconstrained covariance was compared with a model in which the covariance was constrained to equal zero.

**Table 2:** Bivariate GLMM model examining the effect of territory quality on both reactive oxygen metabolites (ROMs) and antioxidant capacity (OXY) simultaneously in the Seychelles warbler. Significant explanatory variables were left in the minimal adequate model after stepwise removal of non-significant variables. Variances and covariances (with standard errors) between the two response variables are given for all random effects

		ROMs			OXY		
			
	d.f.	Estimate ± SE	χ^2^	*P*	Estimate ± SE	χ^2^	*P*
*Final model*							
Intercept		2.38 ± 0.14			138.10 ± 3.52		
Territory quality (log)	1	−0.17 ± 0.07	6.16	0.013	–	–	–
Season^1^	3		107.28	<0.001		45.75	<0.001
2007 NW season		−0.53 ± 0.08			0.31 ± 3.40		
2008 SE season		−0.64 ± 0.07			−17.42 ± 3.15		
2008 NW season		−0.55 ± 0.08			−14.42 ± 3.42		
Time of day	1	−0.001 ± 0.001	3.75	0. 05	0.02 ± 0.01	5.14	0.023
Time of day (squared)	1	<0.001 ± <0.001	7.62	0.006	–	–	–
Breeding stage^2^	3		9.11	0.028	–	–	–
Nest-building		0.10 ± 0.07					
Incubating		−0.09 ± 0.08					
Provisioning		−0.12 ± 0.07					
Sex^3^	1	–	–	–	7.37 ± 2.83	6.78	0.009
Status^4^	2	–	–	–		4.34	0.1 1
Size-corrected body mass	1	−0.06 ± 0.05	0.24	0. 63	−0.15 ± 2.30	0.004	0.95
Sex^3^ × status^4^	2	–	–	–		9.20	0.01
Sex^3^ × helper					−14.86 ± 10.17		
Sex^3^ × non-helper					−16.01 ± 5.63		
*Random effects*							
σ_territory_^2^	1	0.00 ± 0.00	–	–	47.45 ± 27.55	3.45	0.06
σ_individual_^2^	1	0.00 ± 0.00	–	–	42.80 ± 43.28	4.11	0.043
σ_residual_^2^		0.18 ± 0.01	–	–	350.43 ± 45.12	–	–
*Covariance ROM-OXY*							
^cov^territory		–	–	–			
^cov^individual		–	–	–			
^cov^residual		2.00 ± 0.51	1 5.24	<0.001			
*Rejected terms*							
Territory quality (log)	1	–	–	–	−2.70 ± 3.51	0.59	0.44
Breeding stage	3	–	–	–		0.88	0.83
Sex^3^	1	−0.06 ± 0.05	1.46	0. 23	–	–	–
Status	2		0.36	0. 83	–	–	–
Time of day (squared)	1	–	–	–	0.00 ± <0.001	0.06	0.81
Sex × status	2		1.55	0.46	–	–	–

Reference categories: ^1^‘2007 SE season’. ^2^‘pre-nesting stage’. ^3^‘male’. ^4^‘primary’.

Finally, to test separately whether there was an effect of territory quality on body condition, a model was included with body mass as dependent variable and territory, individual and observation as levels. Tarsus length was included as covariate, along with all other biologically relevant terms that were included in the model of Table 1.

## Results

### Territory quality fluctuations

Overall, territory quality was significantly higher in the SE season than in the NW season (GLMM, with territory and observation as random factors, included the terms season: 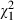 = 46·36, *P* < 0·001, and year: 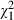 = 0·13, *P* = 0·72). The change from SE to NW season was associated with territory quality changes in most of the coastal territories and in more than half of the inland territories (Fig. 1b). Most of the coastal territories that were shifting from non-wind-exposed to exposed showed a reduction in territory quality (85·7%, *N* = 14). More than half (58·9%, *N* = 24) of the coastal territories shifting from exposed to non-exposed increased in territory quality. Territory quality changes between the seasons are small in many territories, whereas some territories (coastal) undergo large changes (Fig. 1b).

### Territory quality and other factors associated with oxidative status

**Fig. 2:**
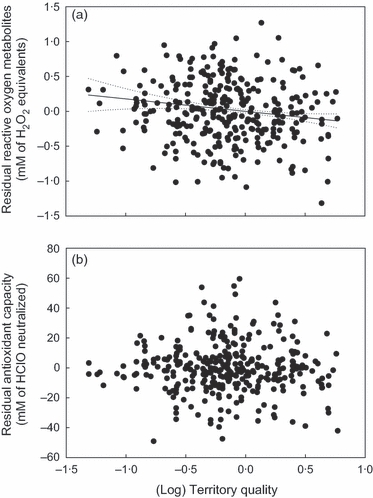
The relationship between territory quality (log-transformed) and (a) ROM levels and (b) antioxidant capacity (OXY) in the Seychelles warbler, presented as residuals of the final models in Table 1. Each point represents one observation (*N* = 339), from a total of 240 individuals. The solid line in (a) represents final model predictions with 95% confidence intervals (dotted lines) based on the model presented in Table 1a.

First, the associations between territory quality (the original predictor effect) and oxidative parameters were investigated. Individuals had significantly higher ROM levels when territory quality was low (Table 1a, Fig. 2a: β = −0·18 ± 0·07, *P* = 0·009). There was no relationship between territory quality and OXY (Table 1b, Fig. 2b).

**Fig. 3:**
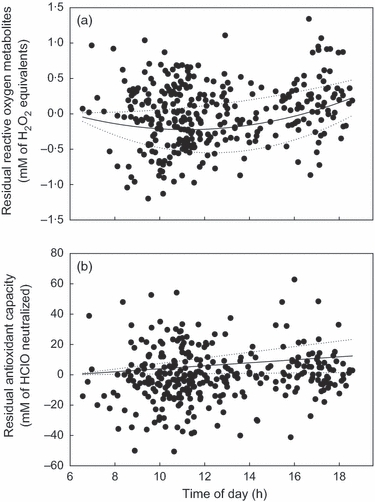
Diurnal patterns of (a) ROM levels and (b) antioxidant capacity (OXY) in the Seychelles warbler, presented as residuals of the final models in Table 1. The solid lines represent final model predictions with 95% confidence intervals.

ROMs and OXY were associated with factors other than territory quality (Table 1a,b). There was a significant effect of breeding activity on ROMs (Table 1a), with levels being higher during nest-building compared to incubation (β = 0·20 ± 0·09, 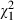 = 5·13, *P* = 0·024) or provisioning (β = 0·23 ± 0·08, 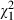 = 7·63, *P* = 0·006). OXY was significantly higher in females than in males, and there was a significant interaction between sex and status (Table 1b). In both sexes, OXY was highest in helpers, followed by primaries and non-helpers, respectively. Particularly in primary birds, patterns of OXY differed between the sexes, with females having significantly higher OXY than males (β = 6·57 ± 2·88, 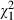 = 5·23, *P* = 0·022). In helpers and non-helpers, patterns were opposite but non-significant. Both ROMs and OXY significantly differed between seasons (Table 1a,b). Particularly, ROMs were notably high in the SE season of 2007, but not in 2008. ROMs were found to be highest very early and very late in the day (quadratic relationship: β = <0·001 ± <0·001, *P* = 0·008, and linear: β = −0·001 ± 0·001, *P* = 0·06; Table 1a; Fig. 3a), and OXY was significantly higher later in the day (linear: β = 0·02 ± 0·01, *P* = 0·022; Table 1b; Figs 3b).

### Within- and between-individual effects

The negative correlation between territory quality and ROMs bordered significance within individuals (β_W_ = −0·23 ± 0·13, 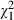 = 3·19, *P* = 0·07) and was significant between individuals (β_B_ = −0·17 ± 0·07, 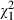 = 5·05, *P* = 0·025). Furthermore, the within- and between-individual effects were effectively the same (β_B_ − β_W_: 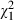 = 0·25, *P* = 0·62) and went in the same direction. Territory quality and OXY were not correlated, either within individuals (β_W_ = −4·23 ± 5·74, 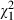 = 0·54, *P* = 0·46) or between individuals (β_B_ = −2·19 ± 3·97, 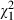 = 0·30, *P* = 0·58). There was no difference between β_B_ and β_W_ effects (β_B_ − β_W_: 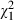 = 0·11, *P* = 0·75), and both effects went in the same direction. As no evidence was found for differences between the within- and between-individual effects of territory quality on either ROMs or OXY, the proceeding analyses used the original predictor variable (i.e. territory quality) without applying within-individual centring.

Within-individual changes in oxidative status were further tested in a subset of repeatedly measured birds living in opposing coastal areas. The shift from the SE to the next NW season resulted in within-individual changes in residual ROMs that went, as expected, in opposite directions (mean change in residual ROMs; birds on the coast shifting from non-exposed to exposed: 0·33 ± 0·22 (*n* = 5) versus birds on the coast shifting from exposed to non-exposed: −0·20 ± 0·17 (*n* = 15); *t* = 1·66, d.f. = 18, *P* = 0·12).

### Oxidative status: integrating ROM and OXY

**Fig. 4:**
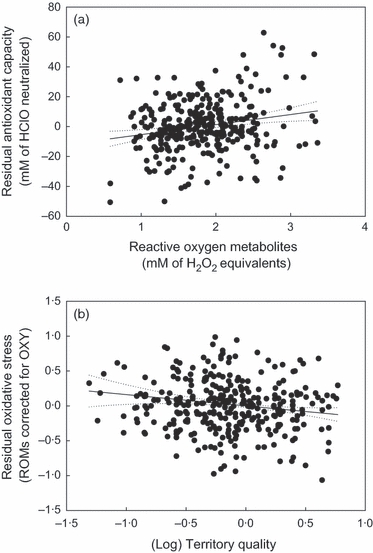
(a) Relationship between ROM levels and antioxidant capacity (OXY) in the Seychelles warbler. OXY is plotted as residuals of a model that included ROMs as independent factor along with all factors present in the previous final models 1a and 1b. The solid line represents the model-predicted response of OXY with 95% confidence intervals. (b) Relationship between territory quality (log-transformed) and oxidative stress. Oxidative stress (calculated as ROMs with OXY as a covariate) is plotted as residuals of a model that included all factors present in the final models 1a and 1b. The solid line represents the model-predicted response of oxidative stress with 95% confidence intervals.

To illustrate the positive correlation between ROMs and OXY (Fig. 4a: β = 11·16 ± 2·57, *P* < 0·001), a GLMM (with territory and individual as random factors) including all factors from the previous final models 1a and 1b was reduced to a final model that included ROMs, season, sex, status, size-corrected body mass and sex × status. A similar three-level structured model was made with ROMs as the dependent variable and OXY as a covariate (thus providing an indication of oxidative stress; see Costantini *et al.* 2008). This model (including all factors present in the final models 1a,b) showed a significant negative association between territory quality and oxidative stress (Fig. 4b: β = −0·16 ± 0·07, *P* = 0·015).

In the bivariate GLMM with both ROMs and OXY included as dependent variables (Table 2), territory quality affected ROMs, indicating that individuals in lower territory quality conditions experienced higher ROMs. The significances of the random terms were as follows: territory level: 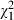 = 2·68, *P* = 0·10 and individual level: 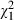 = 4·11, *P* = 0·04. There was no variance in ROMs on the individual or territory level, meaning that there was no repeatability (intraclass correlation) among measurements on these levels. For OXY, repeatability was 0·11 on the territory level and 0·10 on the individual level. Covariance on the lowest (observation) level was positive and significant (within-individual correlation ROM and OXY: r = 0·25; *P* < 0·001, Table 2), whereas covariance on the other levels (territory, individual) was zero, as a result of the lack of variance in ROMs on these levels.

### Body condition

Body condition tended to increase with territory quality (β = 0·20 ± 0·11, 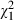 = 3·01, *P* = 0·08) and was significantly influenced by season (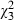 = 13·32, *P* = 0·004), sex (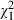 = 61·55, *P* < 0·001) and time of day (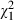 = 11·84, *P* < 0·001). There was a significant interaction effect of sex and breeding stage (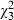 = 22·04, *P* < 0·001).

## Discussion

In the Seychelles warbler, we found a negative association between territory quality and levels of ROMs, but there was no association between territory quality and antioxidant capacity (OXY). ROMs and OXY were positively related, but the relationship between territory quality and ROMs persisted after including OXY as a covariate, indicating that oxidative stress occurs in low territory quality conditions (Fig. 4b). Individual responses to environmental change appear to be important in this system, as shown by both the within-subject centring analysis and the finding that oxidative stress changed between seasons in a subset of repeatedly measured birds.

### Territory quality and ROMs: food availability and salinity effects

Wind-driven salinity could promote oxidative stress in at least two ways. Higher salt intake may directly stimulate oxidant generation and diminish the expression of antioxidant enzymes (Guerriero, Di Finizio & Ciarcia 2002; Lenda & Boegehold 2002; Kitiyakara *et al.* 2003). However, although we find a positive relationship between territory salinity (i.e., through territory quality) and ROM levels in the Seychelles warbler, we find no relationship between territory salinity and antioxidant capacity as would be expected under this scenario. Alternatively, the effect of salinity may be indirect, mediated through the impact of salt on the related biotic environment. Such effects may act either through the organisms that feed on the individual birds (parasites) or through the organisms that the birds feed upon (prey). Previous work in the Seychelles warbler has shown that higher salinity reduces ectoparasite load (Dowling *et al.* 2001), which could indirectly affect the levels of oxidative stress. However, as neither body condition nor survival is correlated with ectoparasite load (Dowling, Richardson & Komdeur 2001), this is perhaps unlikely. In the Seychelles warbler, salinity is likely to have a far greater impact on food availability. On Cousin, wind-borne salt spray defoliates vegetation, resulting in localized reductions in insect availability, and consequently our measure of territory quality (Komdeur & Daan 2005). Reduced food availability can increase oxidative stress in several ways, including the direct stress of poor nutrition on metabolic organs (Robinson *et al.* 1997; Morales *et al.* 2004), the potential psychological stress of food insecurity (Møller, Wallin & Knudsen 1996) and increased metabolic activity accompanying increased foraging effort (Loft *et al.* 1994).

In the Seychelles warbler, individuals in lower-quality territories spend more time foraging for insects from the undersides of leaves while in flapping flight (Komdeur 1991, 1996). Flapping flight is one of the most energetically intensive behaviours a bird can undertake (Nudds & Bryant 2000; Hambly *et al.* 2004), and we would expect greater foraging both to reduce body condition and increase oxidative stress. Body condition did have a tendency to be positively correlated with territory quality (*P* = 0·08). Furthermore, controlling for body condition in the final model of Table 1a did not remove the effect of territory quality on ROMs; though, it did reduce the explanatory power of the territory quality term (Wald statistic decreasing from 9·01 to 6·74). These results suggest that although part of the negative relationship between territory quality and ROM production may be explained by the direct effects of increased salt intake, at least some of the effect was mediated by food availability. The logic being that in low-quality territory conditions, individual ROM levels may be elevated by greater foraging effort as a response to low prey density. Further experiments manipulating food availability and salinity are now needed to separate the direct and indirect effects.

The basis of our argument is that greater foraging effort is related to higher oxygen consumption (Bautista *et al.* 1998; Deerenberg *et al.* 1998; Wiersma, Salomons & Verhulst 2005) and a greater production of oxidants (Loft *et al.* 1994), as seen in honeybees (Williams, Agarwal & Elekonich 2008). However, when making predictions based on metabolic rate as a proxy for oxidant production, one must take care, as these are not necessarily proportional. Much of the support for correlations between metabolic activity and oxidant production comes from studies on organisms unaccustomed to elevated levels of metabolism (Ji 1999; Leeuwenburgh & Heinecke 2001; reviewed in Monaghan, Metcalfe & Torres 2009). Individuals that are used to high levels of metabolism may be able to reduce its oxidative effects through mitochondrial downregulation (e.g., Brand 2000; Speakman *et al.* 2004; Barja 2007) or through enhanced antioxidant defences (e.g., Leaf *et al.* 1999; Leeuwenburgh & Heinecke 2001; Selman *et al.* 2002; reviewed in Monaghan, Metcalfe & Torres 2009). For wild species, such adaptation may occur when high activity is the standard norm (Vianna *et al.* 2001; Brunet-Rossinni 2004; reviewed in Criscuolo *et al.* 2005). Given the exposure of Cousin Island to both abiotic and biotic environmental fluctuations, and the variation in foraging with territory quality (Komdeur 1991), we expect that Seychelles warblers would be accustomed to changes in metabolic activity. Yet we still find greater ROM production in low territory quality conditions and find no differences in antioxidant capacity across different quality territories, suggesting that this species has not been able to evolve to fully avoid such oxidative costs.

### Territory quality and antioxidant capacity (OXY)

We predicted a positive relationship between territory quality and antioxidant capacity (OXY) as birds with access to more food should be able to obtain more dietary antioxidants, leading to improved antioxidant defences and *vice versa*. No such correlation was found, which may imply that food availability is of little importance to antioxidant capacity in this species. Alternatively, the lack of a relationship may be explained by an upregulation of endogenously derived antioxidants to compensate for any shortage of dietary antioxidants during elevated locomotor activity. Such upregulation can arise from increased recruitment, or generation, of endogenous antioxidant components (Vertuani, Angusti & Manfredini 2004) or increased mobilization of dietary antioxidants (Aguiló*et al.* 2005). Antioxidants can be upregulated during exercise (McArdle & Jackson 2000; Radak, Chung & Goto 2005; Gomez-Cabrera, Domenech & Viña 2008; reviewed in Leeuwenburgh & Heinecke 2001), and endogenous antioxidant production can respond to the level of dietary antioxidants ingested (Selman *et al.* 2006; reviewed in Monaghan, Metcalfe & Torres 2009). Hence, endogenous antioxidant regulation may well be a regulatory mechanism designed to prevent oxidative stress (McGraw 2006; Cohen, McGraw & Robinson 2009; Monaghan, Metcalfe & Torres 2009). Such regulatory mechanisms are often difficult to decipher without investigating the full antioxidant barrier, as many antioxidants have multiple roles. For example, uric acid is an antioxidant (Ames *et al.* 1981), but in birds it is also the end product of protein catabolism (Tsahar *et al.* 2006). Further investigations covering the full antioxidant barrier are currently unfeasible in the small endangered species studied here. However, further analyses measuring oxidative damage (lipid peroxidation, protein oxidation) should be conducted to confirm that the imbalance between ROMs and OXY does result in oxidative stress.

Although there may be a biological reason why we find no difference in OXY amongst territories, we cannot exclude the possibility that this result is influenced by the OXY-test not fully representing the total antioxidant capacity. Many assays of antioxidant capacity, based on different antioxidants or free radical substrates, are available. These assays are not necessarily consistent with each other (Cohen, Klasing & Ricklefs 2007; Costantini & Møller 2009), and all have their advantages and shortcomings (see Cohen, Klasing & Ricklefs 2007; Costantini & Møller 2008). The OXY test used here measures a range of antioxidants, but the total antioxidant capacity is not fully covered. First, the test is based on plasma and does not account for antioxidants that occur mainly in cells (e.g., enzymatic antioxidants). Second, the test measures capacity to neutralize HClO, an oxidant of pathologic relevance in biological systems, but it does not reveal the capacity to block other pro-oxidant molecules. We chose the OXY assay for a number of practical reasons. First, it requires only small amounts of plasma – important when working with a small endangered species. Second, it measures the extent to which the antioxidant mechanism can withstand an oxidative attack, rather than quantifying a single antioxidant (Costantini & Møller 2009; Monaghan, Metcalfe & Torres 2009). Third, the contribution of uric acid to the serum antioxidant capacity measured is low (Costantini 2010). Fourth, both oxidative stress parameters (i.e., ROMs and OXY) can be analysed efficiently at the same time, so that repeated freezing/thawing of samples can be avoided. Finally, the OXY test is a commonly used measure that has proven to give valuable information on oxidative status (e.g., Brambilla, Fiori & Archetti 2001; Costantini & Dell'Omo 2006; Costantini, Fanfani & Dell'Omo 2007).

### Oxidative status: integrating ROM and OXY

We investigated the variance in ROMs and OXY at the different grouping levels: (i) territory (i.e., quality); (ii) individual (e.g., genetic quality or phenotypic condition); and (iii) observation (i.e., residual-level variance informative of how temporary changes affect oxidative parameters). We found variance in ROMs only on the observation level, implying that ROMs varied within individuals between observations (as a reaction to their environment or state). There were no territories in which birds had always higher ROMs than in others – possibly because of the alternating winds – and there was no indication that some individuals always had higher ROMs as a result of differences in individual quality. Variance in OXY was also highest on the observation level, but variance at the individual level also occurred, possibly reflecting individual quality differences. Variance on the territory level (for OXY) bordered significance, implying that there was a trend for birds in some territories to always have higher OXY than in others. The significant positive covariance on the observation level indicates that within individuals, higher ROM levels are linked to higher OXY (Fig. 4a), which may be explained by factors other than territory quality.

### Other social and environmental factors associated with oxidative status

Oxidative status was associated with factors other than territory quality (Table 1). For example, there was a positive effect of time of day, quadratic for ROMs and linear for OXY. Diurnal rhythmic changes in pro-oxidants have been hypothesized, perhaps generated by patterns of physical activity (Hardeland *et al.* 2000; Hardeland, Coto-Montes & Poeggeler 2003). Daily patterns have been detected in parameters related to oxidative damage (humans: Bridges *et al.* 1992; Kanabrocki *et al.* 2002; *Drosophila*: Coto-Montes & Hardeland 1999; reviewed in Hardeland, Coto-Montes & Poeggeler 2003), and laboratory studies have reported periodicity of compensatory antioxidant enzymes (reviewed in Hardeland, Coto-Montes & Poeggeler 2003). Therefore, it is possible that daily rhythmicity in both pro-oxidant generation and antioxidant defences may form a basis for an adaptive anticipatory mechanism. However, to our knowledge, this study is the first to report diurnal patterns in pro- and antioxidants in a wild living species; though, a relationship between time of day and plasma carotenoid levels has been found in great tits (*Parus major*; Hõrak *et al.* 2004).

Our investigation also uncovered a seasonal effect on oxidative status, which could be attributed to a large range of variables (e.g., weather conditions). In addition, an effect of breeding stage on ROMs was found, and both a sex-effect and sex-status interactions on OXY were found. These final results suggest that sex-, breeding- and status-related differences in the management of oxidative stress may exist in this species. Further work is now required, focusing on cooperative individuals (*c*. 30% of individuals) to explore the interaction between social status and oxidative stress in the Seychelles warbler.

### Oxidative stress and fitness

Oxidative stress and its management may have considerable effects on fitness. For example, individuals may trade-off allocation of energetic resources into this self-maintenance at the expense of investment into traits like reproduction (Alonso-Alvarez *et al.* 2006; Monaghan, Metcalfe & Torres 2009). Recent studies in the wild have demonstrated such trade-offs between oxidative stress management and fitness-relevant investments such as fecundity (Bizé*et al.* 2008; but see Nussey *et al.* 2009), egg quality (Costantini 2010), growth (Nussey *et al.* 2009) and survival (Bizé*et al.* 2008). Previous research in the Seychelles warbler has shown that lower territory quality results in lower breeding success, because of less time invested in breeding activities and reduced parental care and food provisioning (Komdeur 1991, 1992; but see Brouwer *et al.* 2009). Oxidative stress management may contribute to this pattern, as warblers in low-quality territories have to raise nestlings in poor food conditions, while also battling the damaging consequences of increased oxidant production. Further work is needed to tease apart how different factors related to territory quality affect oxidative stress and fitness parameters in the Seychelles warbler. Translocation of birds from variable quality territories on Cousin to consistently high-quality territories on another island may provide suitable experiments. In a different endangered bird species (hihi, *Notiomystis cincta*), translocation to a novel habitat increased the levels of circulating antioxidants (carotenoids), although food availability was not measured (Ewen *et al.* 2006).

In conclusion, this study indicates that Seychelles warblers individually respond to rapid switches in territory quality. Poor territory conditions lead to increased oxidative stress owing to increased production of ROMs, which is insufficiently counteracted by antioxidant defences. Therefore, predictable variation in oxidative stress experienced by individuals occurs in this natural population, and further studies are needed to investigate the fitness consequences of this variation.
